# Identification of a deep intronic mutation in the COL6A2 gene by a novel custom oligonucleotide CGH array designed to explore allelic and genetic heterogeneity in collagen VI-related myopathies

**DOI:** 10.1186/1471-2350-11-44

**Published:** 2010-03-19

**Authors:** Matteo Bovolenta, Marcella Neri, Elena Martoni, Anna Urciuolo, Patrizia Sabatelli, Marina Fabris, Paolo Grumati, Eugenio Mercuri, Enrico Bertini, Luciano Merlini, Paolo Bonaldo, Alessandra Ferlini, Francesca Gualandi

**Affiliations:** 1 Department of Experimental and Diagnostic Medicine - Section of Medical Genetics, University of Ferrara, Ferrara, Italy; 2 Department of Histology, Microbiology and Medical Biotechnologies, University of Padua, Padua, Italy; 3 IGM-CNR, Unit of Bologna c/o IOR, Bologna, Italy; 4 Department of Child Neurology and Psychiatry, Catholic University, Rome, Italy; 5 Unit of Molecular Medicine, Department of Laboratory Medicine, Bambino Gesu' Hospital, Rome, Italy; 6 Laboratory of Biology, IOR, Bologna, Italy

## Abstract

**Background:**

Molecular characterization of collagen-VI related myopathies currently relies on standard sequencing, which yields a detection rate approximating 75-79% in Ullrich congenital muscular dystrophy (UCMD) and 60-65% in Bethlem myopathy (BM) patients as PCR-based techniques tend to miss gross genomic rearrangements as well as copy number variations (CNVs) in both the coding sequence and intronic regions.

**Methods:**

We have designed a custom oligonucleotide CGH array in order to investigate the presence of CNVs in the coding and non-coding regions of *COL6A1, A2, A3, A5 *and *A6 *genes and a group of genes functionally related to collagen VI. A cohort of 12 patients with UCMD/BM negative at sequencing analysis and 2 subjects carrying a single *COL6 *mutation whose clinical phenotype was not explicable by inheritance were selected and the occurrence of allelic and genetic heterogeneity explored.

**Results:**

A deletion within intron 1A of the *COL6A2 *gene, occurring in compound heterozygosity with a small deletion in exon 28, previously detected by routine sequencing, was identified in a BM patient. RNA studies showed monoallelic transcription of the *COL6A2 *gene, thus elucidating the functional effect of the intronic deletion. No pathogenic mutations were identified in the remaining analyzed patients, either within COL6A genes, or in genes functionally related to collagen VI.

**Conclusions:**

Our custom CGH array may represent a useful complementary diagnostic tool, especially in recessive forms of the disease, when only one mutant allele is detected by standard sequencing. The intronic deletion we identified represents the first example of a pure intronic mutation in *COL6A *genes.

## Background

Mutations in the genes encoding collagen VI (*COL6A1, COL6A2 *and *COL6A3*) result in two major phenotypes: Bethlem myopathy [BM, OMIM #158810] and Ullrich congenital muscular dystrophy [UCMD, OMIM #254090]. Despite BM being classically reported as an autosomal dominant condition due to heterozygous COL6 mutations [1,2], we and others have recently described autosomal recessive BM patients [3,4]. In contrast, the allelic form UCMD was initially considered to be an autosomal recessive disorder, with homozygous or compound heterozygous mutations occurring in all three *COL6 *genes [2], although a few double heterozygous mutations in two different *COL6 *genes have also been described [5]. Recently, however, up to 50% of UCMD cases have been found to carry only one mutated allele, indicating autosomal dominant inheritance [5-7]. Thus far, roughly 100 different mutations in *COL6 *genes have been associated with either UCMD or BM and most of them are confined to single families [5,8].

The distribution of mutations along *COL6 *genes is rather uniform and lacks mutation hot spots, therefore these patients require extensive genotyping, which is currently performed by genomic or cDNA sequencing [1,5]. Nevertheless, a relevant proportion of patients clinically diagnosed as having a collagen-VI related myopathy still lack molecular characterization. In fact, with currently available diagnostic tools, the detection rate of mutations of *COL6 *genes varies from 60-65% in BM cases and 75-79% in UCMD patients [5]. The majority of these mutations are small variations like missense, frame-shifting, ins-del or point mutations which lead to a splicing defect. Large, multi-exon deletions of the *COL6A1 *gene, involving both exonic and intronic regions and thus detectable by mRNA analysis, have been reported as causative mutations in three patients [9,10]. One limitation of PCR-based genome analysis techniques is their inability to detect gross CNVs, as well as atypical mutations, which could account for a significant proportion of undetected *COL6 *mutations. On the other hand, the relatively low rate of mutation detection in *COL6 *genes could be due to the genetic heterogeneity of these diseases. Thus, mutations in genes functionally related to collagen VI could theoretically underlie UCMD and BM phenocopies and/or be responsible for secondary collagen VI defects [11]. In order to test this hypothesis, we selected 12 UCMD/BM patients who were found to be negative upon extensive sequence analysis of the three *COL6 *genes, and two patients carrying only one mutation, deemed insufficient to explain the clinical phenotype, it being inherited from a healthy parent.

The occurrence of both allelic and genetic heterogeneity was explored in these patients by using an innovative oligonucleotide array-based comparative genomic hybridization (CGH) approach able to detect CNVs in *COL6 *genes, as well as in other genes functionally related to collagen VI.

A deep intronic deletion in the *COL6A2 *gene was discovered in one BM patient, with a single mutation inherited from the healthy mother identified at sequencing analysis. Subsequently, the functional effect of the identified mutation was demonstrated via RNA studies. In the remaining patients, only non pathogenic CNVs were identified.

## Methods

### Genome sequence analysis

Patients' genomic DNA was extracted from peripheral blood lymphocytes after informed consent and approval by the local ethics committee was obtained (approval number 7/2009). PCR primers (sequences are available upon request) were designed to amplify all the 107 exons of the *COL6 *genes, as well as their flanking intronic regions. Amplified fragments were directly sequenced using a BigDye Terminator v3.1 Cycle sequencing system on the automated ABI 3130 Genetic Analyzer (Applied Biosystems, Foster City, CA).

**Figure 1:**
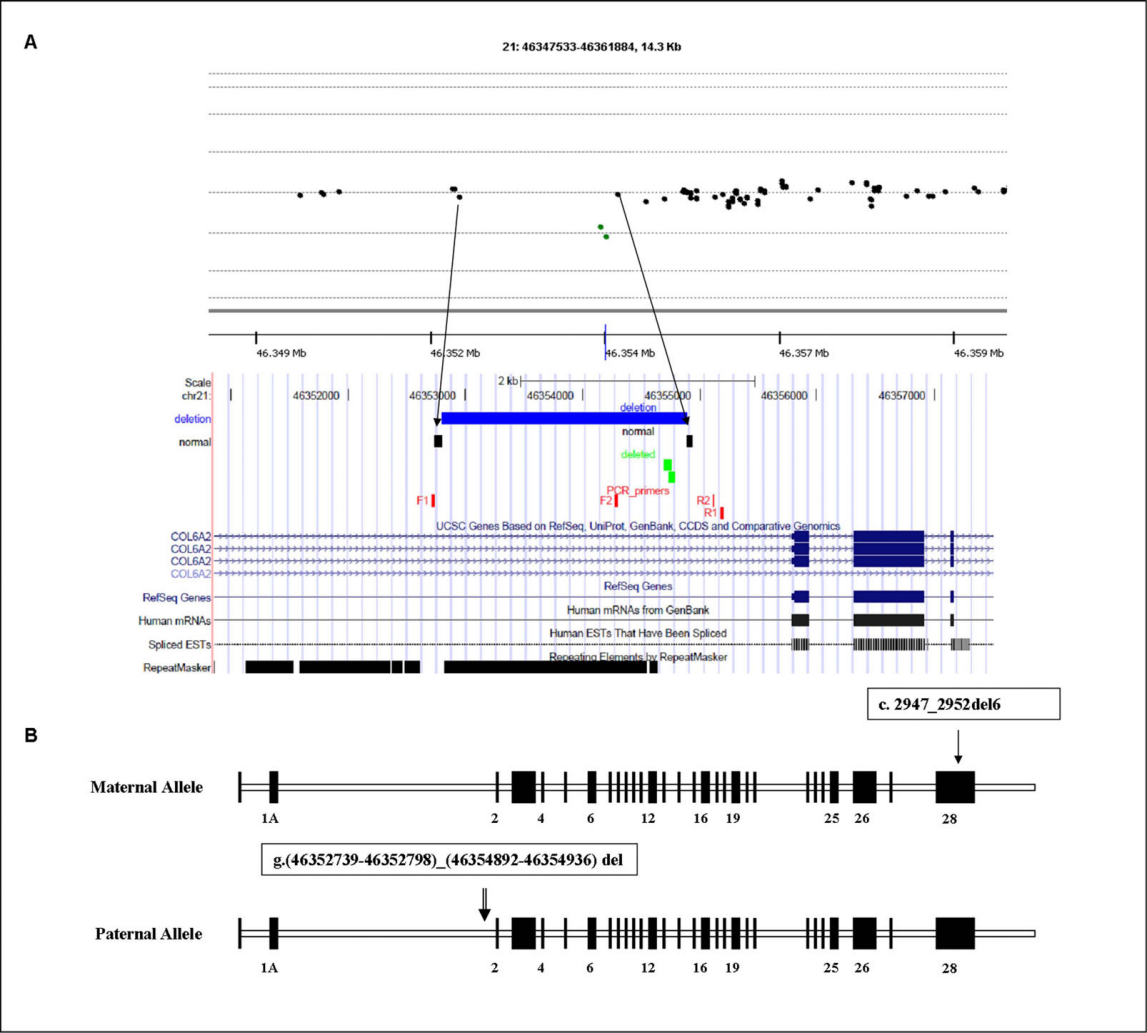
**CGH-array profile and *COL6A2 *gene allelic configuration**. **A)**. CGH-array profile of the *COL6A2 *intron 1A deletion and its schematic representation in the context of the entire *COL6A2 *gene. A custom track containing the maximum theoretical deleted region spanning 2094 bp (blue bar), derived from the closest normal probes (black bars), the two deleted probes (green bars) and PCR primers pairs (red bars), was created on UCSC Genome Browser. The two deleted probes lay at 1054 bp from exon 2. The region between the 5'-normal probe and the first-deleted probe is covered by repetitive elements (black bar at the bottom). The two probes identifying the deletion lye within unique sequences. **B) **Schematic representation of the *COL6A2 *gene allelic configuration in BM Patient 1, with the maternal allele (allele 1) carrying the 6-nucleotide deletion within exon 28 and the paternal allele (allele 2) carrying the intronic deletion within intron 1A.

In order to try to attempt amplification across the deletion within COL6A2 intron 1A, identified by the CGH-array, the following oligonucleotides were positioned outside the maximum theoretical deleted region: F1 CCCTGAATTCCTGGACATGAT; R1 GAACGTCCATCCTCCCTGAT; and flanking the region identified by the two deleted probes (F2 AGATCCACAGCCACGACTT; R2 GGCCTCACTGTGCTGCTG) (Figure 1). Long-range PCR was performed using LA-Taq polymerase and 40 cycles at 64°C.

### Micro-array design, hybridization and data analysis

*COL6*-CGH array design was performed using the high-density aCGH search function of the web-based Agilent eArray database, version 4.5 [12].

The genomic regions corresponding to *COL6A1-A2-A3-A5 *and -*A6 *genes as well as a group of genes functionally related to collagen VI (Table 1) [13-21] were masked for repetitive elements and converted into a 10.197 probe set by selecting the maximum number of exonic and intronic 60 mer oligonucleotide probes available in the Agilent database. This probe set was enriched with 377 probes in triplicate, covering the regions of *COL6A1, 2 *and *3 *genes not investigated by the Agilent CGH probe database. The final mean resolution for these genes was one probe every 320 bp. In order to reach the 15K array format, each array was filled with control probes from all the chromosomes (2851).

**Table 1:** Genes, corresponding genomic regions and protein products included in the *COL6*-CGH micro-array design.

Genes	Chromosomal Coordinates (NCBI Build 35)	Proteins
HSPG2	chr1:21894043-22010053	Heparan sulfate proteoglycan 2
COL6A3	Chr2:237914662-238204820	Collagen, type VI, alpha-3 chain
COL29A1; COL6A6	Chr3:131447049-131978580	Collagen, type XXIX, alpha 1 Collagen type VI alpha 6
ITGA1; ITGA2	chr5:52019893-52526365	Integrin, alpha 1 Integrin, alpha 2
TNXB	chr6:32116611-32186183	Tenascin XB
ITGB1	chr10:33130501-33364492	Integrin, beta 1
DCN	chr12:90041504-90075827	Decorin
ITGA5; ITGA7	chr12:52975314-54487894	Integrin, alpha 5; Integrin, alpha 7
CSPG4	chr15:73654019-73892151	Chondroitin sulfate proteoglycan 4
COL6A1; COL6A2	chr21:46126091-46474147	Collagen, type VI, alpha 1 chain Collagen, type VI, alpha 2 chain
BGN	chrX:152181258-152395851	Biglycan

The array format utilized was 8 × 15K, made up of eight identical 15K arrays on a single slide, thereby permitting simultaneous analysis of eight different samples. Genomic DNA was extracted from the patients' whole blood or cultured fibroblasts by a Nucleon™ BACC Genomic DNA Extraction Kit (GE Healthcare). Labeling and hybridization were performed following the protocols provided by Agilent (Agilent Oligonucleotide Array-Based CGH for Genomic DNA Analysis protocol v5.0). The array was analyzed with the Agilent scanner and the Feature Extraction software (v9.1). A graphical overview and analysis of the data were obtained using the CGH analytics software (v3.5). For identifying duplications and deletions we used the statistical calculations based on ADM-2 algorithm provided by the CGH analytics software. According to this set-up and in the case of autosomal genes, deletions are visualized with values of -1 if in heterozygosity and with values of minus infinite (-4 in CGH analytics) if in homozygosity. For three copies, the value would be approximately +0.6 and for four copies the value would be +1.

The platform informations have been submitted to the online data repository Gene Expression Omnibus (GEO) [22], under accession number GPL9972.

### Real-Time PCR

*Ad hoc *Real-Time PCR assays were designed for the *COL6A2 *intron 1A deletion and the CNVs identified within the ITGB1 and ITGA5/ITGA genomic regions (Table 1 and Additional file 1). For confirming *COL6A2 *intron 1A deletion, a TaqMan assay was firstly used; MGB probe and primer design was performed by Primer Express Software 2.0 (Applied Biosystems) (FW TTGGTCACAGGTTATGCAACA, Rev GGTGAGTTTCACAGCTTCAAGGA; Probe 6-FAM-AACAAGTTAAATAGCATGAAGTG) (see Additional file 2)

Real-Time PCR was performed in triplicate in 96-well plates using 50 ng genomic DNA and default parameters on the Applied Biosystems Prism 7300 system. For relative quantification, the ΔΔCT Method (Applied Biosystems User Bulletin #2) was utilized. *CFTR *exon 15 was employed as a reference gene, and three control DNAs were used as calibrators for each experiment. Specific assays based on SYBR-green chemistry were also employed in order to further verify the intron 1A deletion and to confirm the CNVs identified in the ITGB1 and ITGA5/ITGA7 genomic regions, (Chr10_FW CGTGGAGATGGGATTAGTGTG, Chr10_Rev TTTGTTGGGAATTTACTTGGTG; Chr12_FW AATTTGCTGTTGCTGGGTCT, Chr12_Rev TCCCATACTCTCCATTGTCC; Chr21_FW GCCTGTCTGCCTCTTCCA, Chr21_Rev TGTTGCATAACCTGTGACCAA) (see Table 1 and Additional file 2).

### Transcript analysis in BM Patient 1

Total RNA was isolated from confluent fibroblasts by using an RNeasy Kit (QIAGEN, Chatsworth, CA), and reverse transcribed using a High Capacity cDNA Reverse Transcription Kit (Applied Biosystem, Foster City CA). RT-PCR was performed with a forward primer within *COL6A2 *exon 25 and a reverse primer within the 5' of exon 28, as well as with a forward primer within exon 26 and a reverse within the 3' of exon 28, as previously described [23]. The sequence of primers employed is available upon request.

### Immunohistochemistry, electron microscopy and Western Blot in BM Patient 1

Unfixed frozen sections of the *tibialis anterior *muscle from BM Patient 1 and control were labeled with anti-collagen VI antibody (Chemicon MAB1944) diluted 1:100, followed by FITC-conjugated anti-mouse antibody (DAKO); sections were double-labeled with anti-perlecan antibody (Chemicon) diluted 1:100, and revealed with a TRITC conjugated anti-rat antibody (SIGMA). Muscle sections were also labeled with anti-caveolin 3 (BD-Transduction), collagen IV, laminin α2 and laminin β1 chains (Chemicon), followed by FITC-conjugated secondary antibody, while fibronectin (Sigma) and alpha-dystroglycan (Upstate Biotechnologies) were followed by TRITC-conjugated antibody (Sigma, MO). The fibroblast cultures from Patient 1 and from a control were obtained by mechanical means from bioptic skin fragments and set up as previously described [24]. A mouse monoclonal anti-collagen VI antibody (MAB1944, Chemicon) was employed, and the resulting immunoreaction was detected with a secondary FITC-conjugated anti-mouse antibody (DAKO). For immunoelectron microscopy analysis, cells were incubated with a monoclonal anti-collagen VI antibody (Chemicon) diluted 1:25 with Dulbecco's modified Eagle's medium and with 5 or 15-nm colloidal gold-labeled IgG (Amersham). Rotary shadowing electron microscopy was performed as described [25].

Samples derived from skin fibroblast cultures and muscle biopsies were prepared for Western Blot analysis as previously described [23]. Collagen VI was detected by immunoblotting with antibodies recognizing either all collagen VI chains (Fitzgerald 70XR95) or the α1(VI) chain (Santa Cruz sc-20649) alone. Antibodies recognizing AKT (Cell Signaling) and myosin (Sigma) were used for cell culture loading and muscle samples.

## Results

### Patient selection and COL6-CGH array validation

Twelve patients clinically diagnosed as possessing UCMD (6 patients) or BM (6 patients) phenotypes negative at genomic sequence analysis, and 2 patients (1 UCMD, 1 BM) in whom a sequence analysis positive for *COL6 *mutations failed to fully explain the clinical phenotype were selected (Table 2).

**Table 2:** Summary of clinical and genomic data in the analyzed BM/UCMD patients.

SAMPLE ID	COL6A1-A2-A3 Genomic Sequencing	Inheritance	Sex	Presentation	Age at review, yr	Max motor ability (Power Grade)	Contractures	Distal laxity	Skin phenotype	Creatine kinase level*	Respiratory function (% of predicted)	Cardiac evaluation	Collagen VI SKM	Array Results
1 (BM) [26]	COL6A2 c.2947_2952del 6 het (mat)	Sporadic	F	3 yrs, difficulty in running & climbing stairs	20	Walked 15 mo-present	Neck, elbows, fingers, knees, ankles	Fingers	Rough skin, dystrophic nails, cheloids	3.5	52%	normal	Mildly reduced at BM of several muscle fibers	chr21 g.(46352739-46352798) _ (46354892-46354936) del
2 (UCMD)	COL6A1 c.350 C>T het p.V117A (pat)	Sporadic	M	Birth, hip dislocation	4	Able to walk 4 UL and 3,4 LL	Knees, ankles	Fingers	none	1.5	89%	normal	ND	-
3 (BM)	-	Familial	F	3 yrs, difficulty in running & climbing stairs	48	Walked 13 mo-present	Neck, elbows, fingers, ankles	none	none	2	58%	normal	ND	-
4 (BM)	-	Familial AD	M	3 yrs, unable to collect objects from the floor	24	Walked 12 mo-present	Neck, elbows, fingers, knees, ankles	Fingers	Hypertrophic scars	1.5	74%	normal	Mildly reduced at BM of some muscle fibers	chr10 g:(33176784_33176843) _(33178291_33178350)del
5 (BM) [45]	-	Familial AD	M	18 yrs, reduced stamina	36	Walked 12 mo-present	Neck, fingers, knees, ankles	none	none	4	95%	normal	Normal amount and localization	chr12 g:(54068784_54068843) _(54070617_54070676)dup
6 (BM)	-	Sporadic	M	5 yrs, difficulty in bending forward	15	Walked 12 mo-14 yrs	Neck, trunk, elbows, fingers, hips, knees, ankles	Fingers	none	18	44%	normal	Normal amount and localization	-
7 (BM)	-	Familial AD	M	30 yrs, easily fatigued, falls	60	Walked 12 mo-present	Fingers, ankles	Fingers	none	2.5	ND	normal	Normal amount and localization	-
8 (BM)	-	Familial AD	F	5 yrs, contractures	39	Walked 12 mo-present	Shoulders, elbows, fingers, hips	None	none	0.8	ND	Sinus tachycardia	ND	-
9 (UCMD)	-	Sporadic	F	Birth with pes talus	3	Walked 14 mo-present	none	Fingers	none	2	ND	ND	Reduced at BM of muscle fibers	-
10 (UCMD) [11]	-	Sporadic	F	Birth with delay in motor milestones	8	Able to walk 4 UL and LL	none	Fingers	Follicular hyperplasia	normal	85%	normal	Reduced at BM of muscle fibers	-
11 (UCMD)	-	Sporadic	M	2 yrs, hyperlaxity and hyperCK	8	Able to walk 1 hip flex - 3 knees ext	Fingers, hip, knees, ankles	None	none	2	33%	normal	ND	-
12 (UCMD)	-	Sporadic	F	18 mo, unable to walk	31	Able to walk 24 mo-12 yrs	Neck, trunk, elbows, fingers, hips, knees, ankles	None	none	1.5	62%	normal	Normal amount and localization	-
13 (UCMD)	-	Sporadic	F	Birth, floppiness	3	Walked 18 mo-present	Fingers, knees, ankles	Fingers	Follicular hyperplasia	2	ND	ND	Reduced at the BM of muscle fibers	-
14 (UCMD)	-	Sporadic	M	Birth	4	Able to walk at 3 yrs	Congenital kyphosis	Fingers	Follicular hyperplasia	normal	ND	normal	ND	-

*Creatine kinase level: times the upper value of normal.

UL: upper limbs; LL: lower limbs; AD: autosomal dominant; yrs: years; mo: months; ND: not done; SkM: skeletal muscle; BM: basement membrane

One BM patient (Patient 1) had previously been described by Demir *et al*. (# 10, Family 8), who linked the disease in this family to chromosome 21q22.3 [26]. This patient carries a heterozygous small deletion in exon 28 of the *COL6A2 *gene (NM_001849, c. 2947_2952del6, p.Asp983_Val984del) inherited from the healthy mother. Patient 2 (UCMD) bears a heterozygous missense alteration (NM_001848, c.350 C>T, V117A) within *COL6A1 *exon 3, inherited from the unaffected father. The V117A substitution noted in this patient had not previously been described, so in order to evaluate the pathogenic effect of this missense variation, polymorphism phenotyping predictions were obtained by PolyPhen [27] and SIFT [28] with contrasting results: PolyPhen predicted a benign variation whereas SIFT foresaw a harmful effect of the V117A substitution. Subsequently, amino acid conservation of V117 was analyzed, and sequence alignment between distant species showed high conservation of the residue in the VWFA1 domain (data not shown). The screening of 200 control chromosomes excluded it as a common polymorphism.

The COL6-CGH array was designed to cover the regions of genes *COL6A1-A2-A3-A5 *and -*A6 *and a group of genes functionally related to collagen VI (Table 1). Ten possible candidate genes were considered on the basis of the following criteria: either i) their direct interaction with collagen VI, as in the case of HSPG2 [13], ITGA1, ITGA2, ITGB1 [14], DNC and BGN [15]; ii) their secondary involvement in collagen VI-deficient tissues, as in NG2-proteoglycan [16,17], ITGA5 and ITGA7 [18,19], or iii) their mutations causing an overlapping phenotype with collagen VI-related myopathies, as in TNXB [20]. COL6A5 (COL29A1) and COL6A6, which are expressed in skeletal muscle, were also included since collagen VI alpha1-deficient mice do not express collagen VI alpha 5 or alpha 6 chains [21].

The full coding region of all selected genes was included, together with intronic sequences and 100 kb at the 5' and 3' of the first and last exons, respectively. The array was technically validated before commencing by using DNA from 8 normal controls; no CNVs were detected.

Two novel CNVs were identified in BM patients 4 and 5 (Table 1 and Additional File 2). The first was a 1.4 Kb deletion, located 35 Kb downstream the ITGB1 gene and the second was a 1.7 Kb duplication in the intergenic region between ITGA5 and ITGA7. Both CNVs were confirmed by Real-Time PCR analysis (data not shown), but the occurrence of both variations in unaffected family members and healthy controls from the general population permitted exclusion of their pathogenic significance (see Additional File 2).

The complete CGH datasets have been submitted to the online data repository Gene Expression Omnibus [22], under accession number GSE20025.

### Identification of a pure intronic deletion in intron 1A of the COL6A2 gene in BM patient 1

In early infancy, Patient 1 (BM) (Table 2) attained normal motor milestones and walked at 15 months of age. However, aged 3 and a half, she began to have difficulty running. Creatine kinase was 3.5 times the upper limit of normal, and examination at age 21 revealed a short stature, excessive weight and bilateral club foot. The patient was able to walk and climb stairs without support, but was unable to run. Mild facial and limb/girdle weakness and moderate axial and distal weakness were noted. The patient was unable to bury the eyelashes completely or to flex neck against gravity, and digitorum extension weakness was evident. The patient suffered contractures of the neck, elbows, fingers, knees and ankles, combined with finger extension hypermobility when the wrist was flexed. Her skin was rough and cheloidal, and nails were dystrophic. Forced vital capacity was 52%, and cardiac examination evidenced no abnormality. Examination at age 30 years revealed a relatively stable condition, with only the ability to get up from the floor having been further compromised.

In BM Patient 1, CGH analysis revealed the presence of a deletion within intron 1A of the *COL6A2 *gene (NM_001849). This deletion was detected by two overlapping probes which covered 95 base pairs: the 5'-deleted probe lying 12.237 nucleotides from exon 1A, and the 3'-deleted probe located 999 nucleotides from exon 2. The two flanking probes, which showed normal hybridization levels, were localized respectively, 1900 nucleotides from the proximal 5'-deleted probe and 194 nucleotides from the distal 3'-deleted probe. Thus, the identified deletion spans a maximum theoretical region of 2094 bp, and is located 1 kb upstream of the first *COL6A2 *coding exon (exon 2). Comparative sequence analysis using the two deleted probes identified a genomic region lying adjacent to a cluster of repetitive elements (Figure 1A) [29].

Real-Time PCR analysis performed using two different assays, one based on SYBR-green and the other on TaqMan chemistries, confirmed the occurrence of the deletion, which was inherited from the healthy father (see Additional File 3). Analysis of 100 control subjects from the normal population by Real-Time PCR failed to identify the *COL6A2 *intron deletion.

Genotypic analysis of the proband's parents showed the intronic deletion and the exon 28 small mutations in *trans*, denoting autosomal recessive transmission.

An attempt to amplify by junction PCR the deletion breakpoint with primers outside the maximum theoretical deleted region was unsuccessful, and sequencing analysis of the PCR products obtained using different sets of primers failed to identify the mutated allele. This suggests the possibility of a complex rearrangement/inversion of the involved genomic region (Figure 1A).

### Occurrence of monoallelic COL6A2 transcription in BM Patient 1 fibroblasts

**Figure 2:**
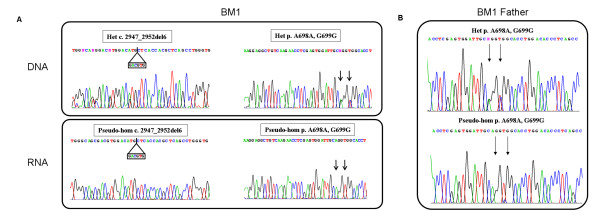
***COL6A2 *genomic and transcript analysis in BM Patient 1 and his father**. **A) **Sequence chromatograms of BM Patient 1 showing the maternal deletion within exon 28 (GACGTG) (left) and two polymorphisms (c.2094 G>A - A698A; C.2097 C>T - G699G) within exon 26 (black vertical arrows) (right) occurring heterozygously at the DNA level (upper panel) and pseudo-homozygously at the RNA level (lower panel). **B) **Sequence chromatograms in the BM Patient 1 father showing that the exon 26 polymorphisms (c.2094 G>A - A698A; C.2097 C>T - G699G) occur heterozygously at the DNA level (upper panel). At the RNA level (lower panel) the nucleotide variants that are undetectable in the BM Patient 1 (c.2094 G and c.2097 C), are only barely visible in the father.

In order to assess the pathogenic meaning of the identified intronic deletion found in BM Patient 1, fibroblasts were harvested and cultured prior to RNA analysis. This analysis was focused on the terminal region of the *COL6A2 *transcript, from exon 26 (harboring two common polymorphisms (Ala698Ala and Gly699Gly), heterozygous at the genomic analysis) to exon 28 (the site of the maternal heterozygous 6-nucleotide deletion). Direct sequencing of the amplified fragments revealed the presence of a pseudo-homozygosity for the exon-28 deletion, as well as for the polymorphisms within exon 26 (Figure 2). This implies that the allele carrying the intronic deletion is not transcribed at appreciable levels.

In order to strengthen the case for a relationship between the identified intronic deletion and the transcriptional behavior, RNA analysis was performed on cultured skin fibroblasts from the patient's father. The two exon 26 polymorphisms (Ala698Ala; Gly699Gly) were found to be heterozygous at the genomic level, although their transcription was strongly imbalanced. The nucleotide variant of the polymorphisms (c.2094 G>A - A698A; C.2097 C>T - G699G) that was completely undetectable in the proband's RNA (G and C, respectively), was only barely visible in cells from the father (Figure 2).

### Collagen VI expression in muscle and cell cultures from BM Patient 1

**Figure 3:**
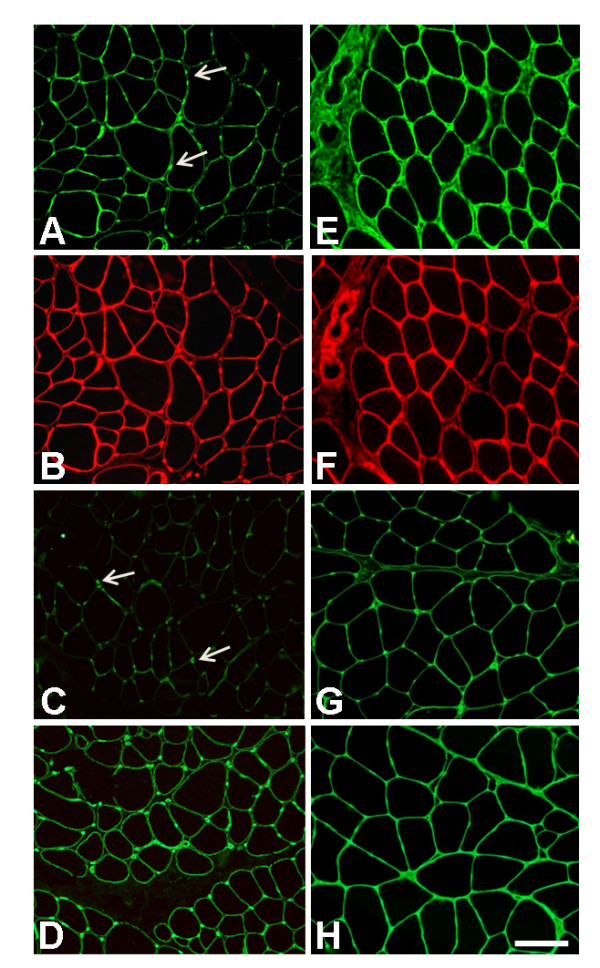
**Immunohistochemical analysis on muscle biopsy from BM patient 1**. Immunofluorescence analysis on muscle sections of BM Patient 1 (**A-D**) and control (**E-H**) of collagen VI (**A, E**), perlecan (**B, F**), laminin β1 (**C, G**) and collagen IV (**D, H**). A small reduction in collagen VI in the patient's endomysium (**A**) was observed in comparison with control (**E**). However, collagen VI was expressed normally around the blood vessels (arrows, **A**). Double-labeling with anti-perlecan antibody revealed a normal pattern (**B**) as well in the control section (**F**). Laminin β1 expression was reduced at the basal lamina of muscle fibers, while being expressed normally around the capillary walls (arrows, **C**). Collagen IV labeling showed a normal pattern around both vessels and muscle fibers (**D**). Bar, 40 μm.

**Figure 4:**
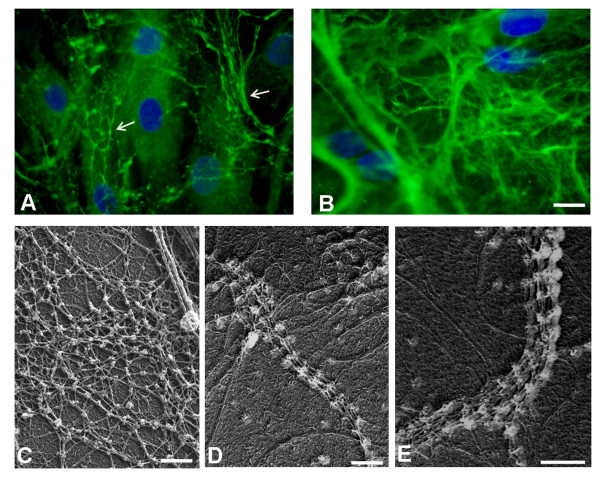
**Immunohistochemical analysis and electron microscopy on fibroblasts**. Reduced amount of collagen VI microfilamentous network was detected in the extracellular matrix secreted by fibroblasts of BM Patient 1 (**A**) as compared to control fibroblasts (**B**). The microfibrillar network in the patient's fibroblasts showed an altered organization, characterized by a coarser texture than normal (**A**, Arrows) Fibroblast density was monitored by DAPI staining of the nuclei. Bar, 20 μm. Electron microscope examination of rotary-shadowed cultured fibroblasts from control (**C**) and BM Patient 1 (**D-E**), labeled with anti-collagen VI and revealed with a secondary antibody conjugated with 5-nm colloidal gold particles. Ultra-structural analysis of the rotary-shadowed fibroblasts revealed the presence of collagen VI fibrils constituted by several parallel running microfilaments in BM Patient 1 (**D, E**), while regular webs of interconnected microfilaments, like those shown in control fibroblasts (**C**), were absent. Bar, 200 nm.

**Figure 5:**
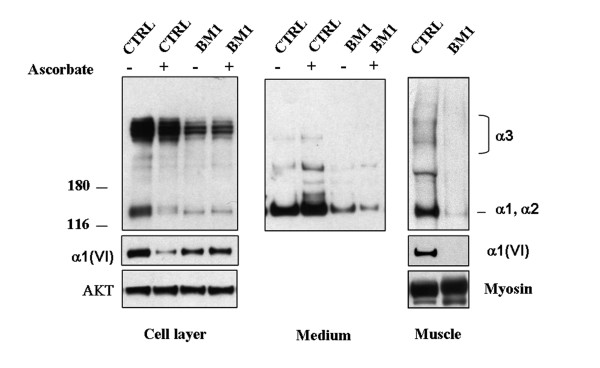
**Western Blot analysis of fibroblasts**. Western blot analysis of collagen VI in cultured skin fibroblasts and in muscle biopsies from control (CTRL) and BM Patient 1 (BM1) was performed. Samples corresponding to the cell layer (left panel, 20 μg) and medium (middle panel, 30 μg) in the presence or absence of L-ascorbate, and muscle extracts (right panel, 15 μg), were separated by electrophoresis under reducing conditions in 3-8% polyacrylamide-gradient gel. Collagen VI was detected by immunoblotting with antibodies recognizing either all collagen VI chains (Fitzgerald 70XR95) or the α1(VI) chain (Santa Cruz sc-20649). Migration of the collagen VI chains is indicated on the right and molecular weight markers (kDa) are shown on the left. Control for loading was performed by antibodies against AKT (cell layers) or myosin (muscle biopsies).

Muscle biopsy of Patient 1 revealed a mild reduction in collagen VI in the endomysium (Figure 3A) in comparison with control (Figure 3E). Double labeling with anti-perlecan antibody showed a normal pattern, attesting the integrity of the basement membrane. Alpha-dystroglycan, caveolin 3, fibronectin, and laminin α2 chains (data not shown) were normally expressed, as was collagen IV (Figure 3D), while laminin β1 chain labeling was reduced around the muscle fibers and preserved at the basement membrane of blood capillary vessels (Figure 3C). In cultured skin fibroblasts, a mildly reduced expression of collagen VI protein was associated with an altered organization of the microfibrillar network: the immunofluorescence pattern was characterized by a coarser texture than normal with fewer thinner fibrils (Figure 4A). A finely structured collagen VI network was no longer visible, while thicker fibers were still present. Electron microscopy analysis of rotary-shadowed replicas of patient's *in vivo*-labeled fibroblasts showed the presence of thick collagen VI fibrils, constituted by several parallel microfilaments, while regularly developed webs of interconnected and cross-linked filaments like those seen in control fibroblasts were absent (Figure 4C-E). Western blot of fibroblast cultures and muscle biopsy samples using two different antibodies recognizing either all collagen VI chains or the α1(VI) chain alone revealed a quantitative deficiency of collagen VI in the patient, as compared to an unaffected control (Figure 5). The reduced amount of collagen VI in skeletal muscle was confirmed by immunoblotting for myosin, used as a loading control for normalizing the amount of muscle tissue.

## Discussion

Molecular genotyping of UCMD and BM patients is currently performed by extensive sequencing of *COL6A1, A2 *and *A3 *genes. Unfortunately, however, the PCR-based techniques used in routine screening miss gross rearrangements as well as CNVs which exceed the dimensional limitations of PCR amplification. Furthermore, molecular analysis fails to identify the causative mutation in a significant percentage of patients, ranging from 20-25% in UCMD to 35-40% in BM [5]. This relatively low detection rate of current molecular approaches in collagen VI myopathies could be ascribed to allelic heterogeneity (linked to the abovementioned limitations of PCR-based techniques) and/or genetic heterogeneity. This latter hypothesis implies that UCMD/BM phenocopies could occur, due to mutations in different, still unidentified genes.

In the last few years, novel genomic-based technologies have been reported as an efficient and improved alternative to PCR in molecular diagnosis. Oligonucleotide array-based CGH was initially developed to detect major changes in chromosomal copy number [30], and since then both commercial and custom arrays have been also used to discern these changes in selected genomic regions of interest [31,32]. The *DMD *gene was a perfect candidate to test the validity of this approach, due to the large size and high number of gross copy number variations apparent in this condition. Moreover, different custom arrays have already been validated for the exploration of both coding [33] and non-coding regions of the gene [34-36]. For *COL6 *genes, the possibility that genomic dosage imbalance or large CNVs occur with significant frequency in UCMD/BM patients still remains untested.

With the aim of increasing the sensitivity of molecular diagnosis in collagen VI-related disorders, a custom oligonucleotide-based CGH array able to detect CNVs in coding and non-coding regions of the *COL6A1, A2, A3*, and the *A5 *and *A6 *genes recently discovered to encode novel collagen VI chains [21,37] was designed. Moreover, in order to investigate genetic heterogeneity, the genomic regions of other genes selected on the basis of their known or hypothetical functional relationship with collagen VI were also included in the design, as all these genes potentially represent candidates for mutations that could underlie phenocopies of collagen VI-related diseases [38,13,15-42].

By testing our array in 14 selected UCMD/BM patients, we identified a deep intronic deletion in the *COL6A2 *gene in a BM patient occurring in compound heterozygosity with a small exonic mutation previously detected by sequencing. Despite not definitively proven, due to the failure to amplify the deletion breakpoint, nevertheless the pathogenic potential of this intronic mutation is supported by the transcriptional impairment of the mutated allele that was demonstrated both in the proband and in her healthy carrier father. The *COL6A2 *gene is characterized by a first-coding exon (exon 2) separated from two alternatively spliced 5'-untranslated exons (exon 1 and 1a) by a huge 12 kb intron (intron 1A) [43].

Deletion of this intron may abolish *cis*-acting elements and/or the binding of *trans*-acting factors involved in the regulation of *COL6A2 *gene expression. Alternatively, the deletion itself might be the marker for a complex genomic rearrangement occurring in the *COL6A2 *gene that inverts or scrambles the entire genomic configuration. The failure of PCR to amplify the deletion junction seems to support this hypothesis.

This study documents an additional case of a BM patient with a compound heterozygous genotype for recessive *COL6A2 *mutations. Interestingly, both of the autosomal recessive BM cases we previously described carried a peculiar allele combination consisting of a truncating mutation partnered by missense changes within the α2(VI) C2 region [3]. Despite different (null mutation/in-frame deletion), the allelic configuration of BM Patient 1 also indicates the presence of a mutated α2(VI) C2 domain, derived from a single allele, similar to the recessive BM cases previously described [3]. The findings in this patient also substantiate the observation that recessive BM mutations, unlike the classical dominant cases, affect collagen VI expression in skeletal muscle [3], as attested by immunohistochemical and biochemical analyses showing a decreased amount of this protein.

In the twelve patients who tested negative upon genomic sequence analysis, no CNVs were identified in *COL6 *genes using the CGH array. Even though the limited size of the analyzed patient cohort hampered definitive estimations, our results did exclude a relevant incidence of CNVs within *COL6 *genes, thereby supporting UCMD/BM genetic heterogeneity. Likewise, no pathogenic CNVs were identified among the other genomic regions potentially harboring candidate BM/UCMD genes that were explored in the array. Nevertheless, these results do not rule out the possibility that genetic heterogeneity could account for some *COL6A1-3*-negative patients and suggest that high-throughput sequencing technologies could represent more appropriate future approaches for detection of point mutations. In fact, these innovative tools could allow significant enlargement of the spectrum of functionally related collagen VI genes to be explored sequentially via CNV identification and re-sequencing for detection of point mutations.

## Conclusions

The described COL6-CGH array could represent a useful complementary diagnostic test, useful for increasing the sensitivity of molecular analysis in patients with a clinical diagnosis of collagen VI-related disorders. The limited size of the patient cohort we analyzed hampered estimation of copy number variation frequency, but analysis of larger populations could well permit conclusions to be drawn. In fact, this novel tool allowed us for the first time to identify a *COL6A2 *mutation affecting gene transcription deeply located within an intronic region, and thus undetectable with all other techniques currently available. Furthermore, in recent years specific genetic diagnosis has become mandatory for a patient to be eligible for upcoming therapeutic trials [44], and thus the lack of molecular diagnosis in a large percentage of patients with collagen-VI related phenotypes makes the search and the validation of novel diagnostic tools an ever-more pressing issue.

## Competing interests

The authors declare that they have no competing interests.

## Authors' contributions

MB designed the array and carried out DNA extraction and hybridization of samples, Real-time PCR experiments, and analysis and interpretation of data; MN performed DNA extraction and hybridization of samples, Real-time PCR experiments, and contributed to preparation of the manuscript; EM performed genomic and transcript analyses; MF prepared fibroblasts cell cultures; AU and PG carried out Western Blotting; PS performed ICC analysis; EM and EB carried out clinical evaluation of the patients; LM clinically evaluated the patients and participated in revision of the manuscript; PB assisted in the interpretation of data and revision of the manuscript; AF contributed to conception of the study and preparation of the manuscript; FG conceived and designed the study, prepared and revised the manuscript and approved the final version.

## Pre-publication history

The pre-publication history for this paper can be accessed here:

http://www.biomedcentral.com/1471-2350/11/44/prepub

## Supplementary Material

Additional file 1 Gene Symbols, chromosomal coordinates, transcript and protein identifiers for the genes included in the *COL6*-CGH micro-array design. Click here for file

Additional file 2 **CNVs identified in BM Patients 4 and 5**. **A) ***COL6*-CGH array result in BM Patient 4, showing the deletion of about 1.4 kb identified on chromosome 10, 35 Kb downstream of the ITGB1 gene. **B) **The CNV on chromosome 10 was validated by Real-Time PCR, and its segregation was analyzed in Patient 4's family; the deletion was present in three unaffected subjects (2-ΔΔCT values of 0.44, 0.41, 0.45) and absent in the symptomatic proband's mother and cousin (2-ΔΔCT values of 0.99 and 0.86), thus not linked to the disease. **C) ***COL6*-CGH array result in BM Patient 5, showing the 1.7 Kb duplication occurring in the intergenic region between ITGA5 and ITGA7 on chromosome 12. Click here for file

Additional file 3 **Real Time PCR experiments confirming COL6A2 intron 1A deletion in BM Patient 1 and in the father**. In the upper panel, the results obtained with an intron 1A specific TaqMan assay are shown. Red plots correspond to utilized reference gene (CFTR exon 15) whereas green plots refer to target sequence within intron 1A. The Ct (threshold cycle) values of the target sequence are in line with the reference in control sample (unaffected subject) and in proband's mother, whereas the target Ct values are higher than reference in BM Patient 1 and in the father, attesting the deletion (2-ΔΔCT values were 0.47 and 0.53 in BM Patient 1 and in the father respectively, whereas the 2-ΔΔCT value was 0.97 in the proband's mother). In the lower panel the position of the primers utilized in the SYBR green assay (blu) and of primers and probe utilized in the TaqMan assay (pink), are shown in respect to the deleted region (in bold). Click here for file
