# Supplementary figures and images for: Identification of a deep intronic mutation in the COL6A2 gene by a novel custom oligonucleotide CGH array designed to explore allelic and genetic heterogeneity in collagen VI-related myopathies
Source: BMC Med Genet. 2010 Mar 19;11:44. doi: 10.1186/1471-2350-11-44 (PMC2850895; doi:10.1186/1471-2350-11-44)

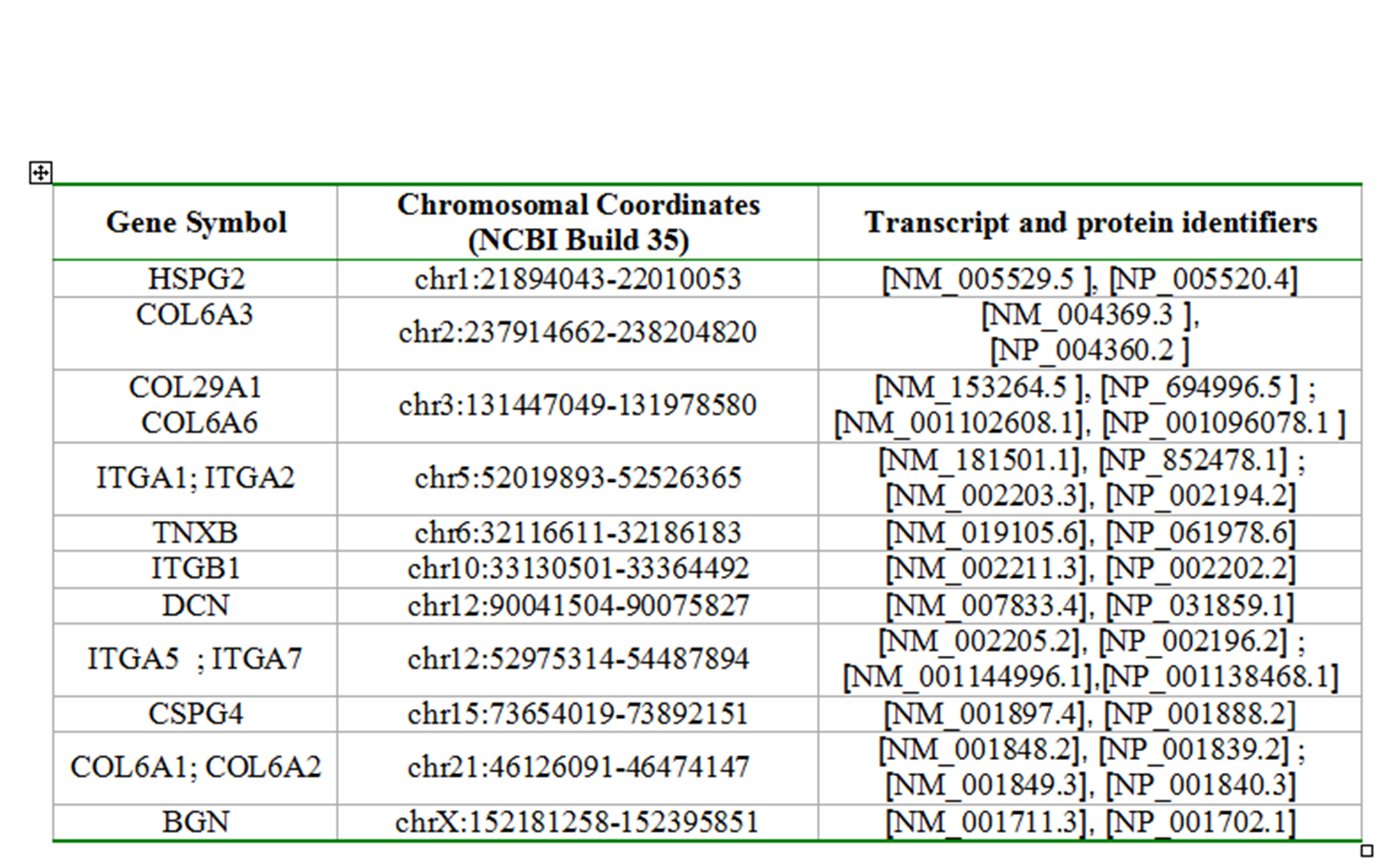

Supplement: Additional file 1 — Gene Symbols, chromosomal coordinates, transcript and protein identifiers for the genes included in the COL6-CGH micro-array design. [file 1471-2350-11-44-S1.PNG]

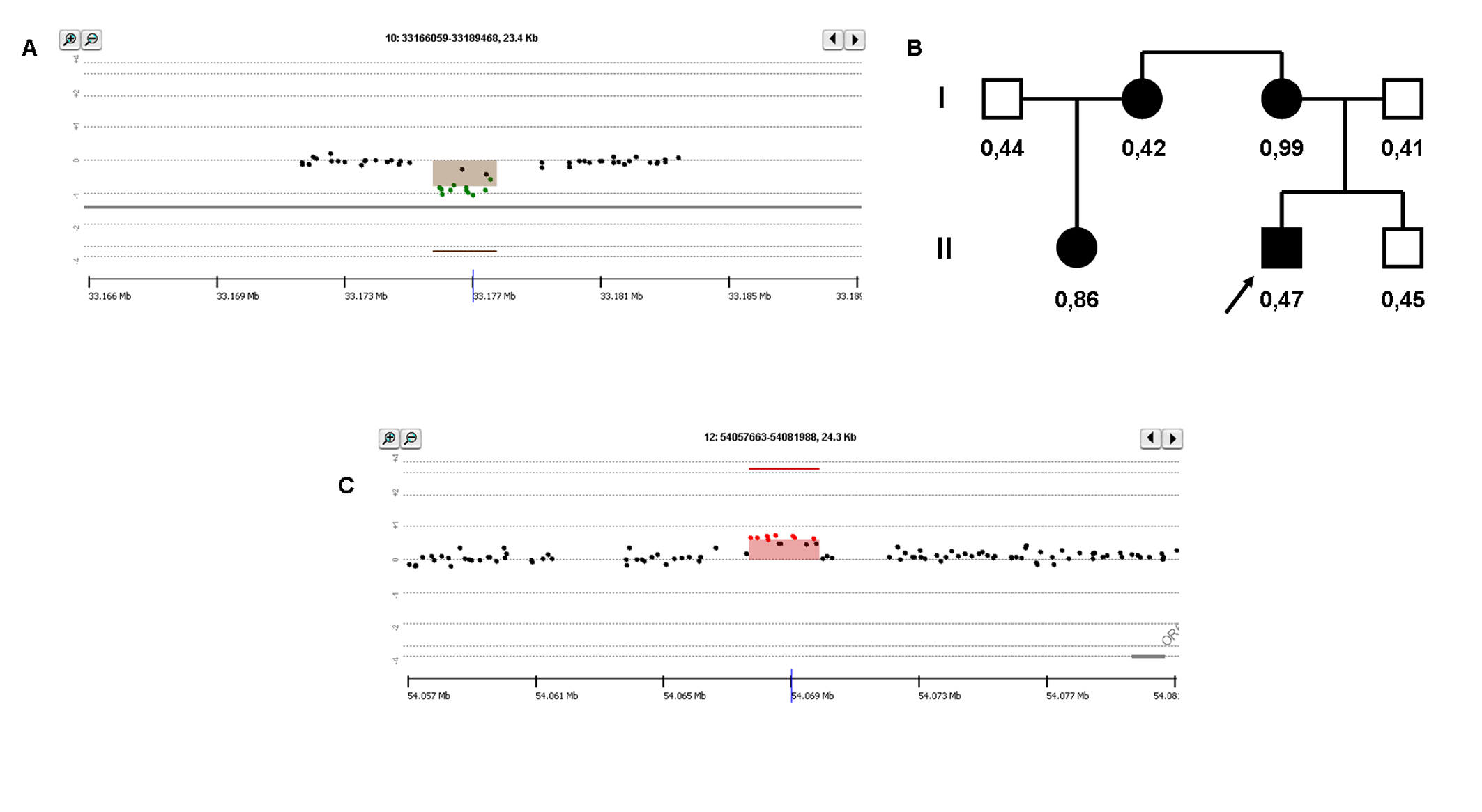

Supplement: Additional file 2 — CNVs identified in BM Patients 4 and 5. A) COL6-CGH array result in BM Patient 4, showing the deletion of about 1.4 kb identified on chromosome 10, 35 Kb downstream of the ITGB1 gene. B) The CNV on chromosome 10 was validated by Real-Time PCR, and its segregation was analyzed in Patient 4's family; the deletion was present in three unaffected subjects (2-ΔΔCT values of 0.44, 0.41, 0.45) and absent in the symptomatic proband's mother and cousin (2-ΔΔCT values of 0.99 and 0.86), thus not linked to the disease. C) COL6-CGH array result in BM Patient 5, showing the 1.7 Kb duplication occurring in the intergenic region between ITGA5 and ITGA7 on chromosome 12. [file 1471-2350-11-44-S2.PNG]

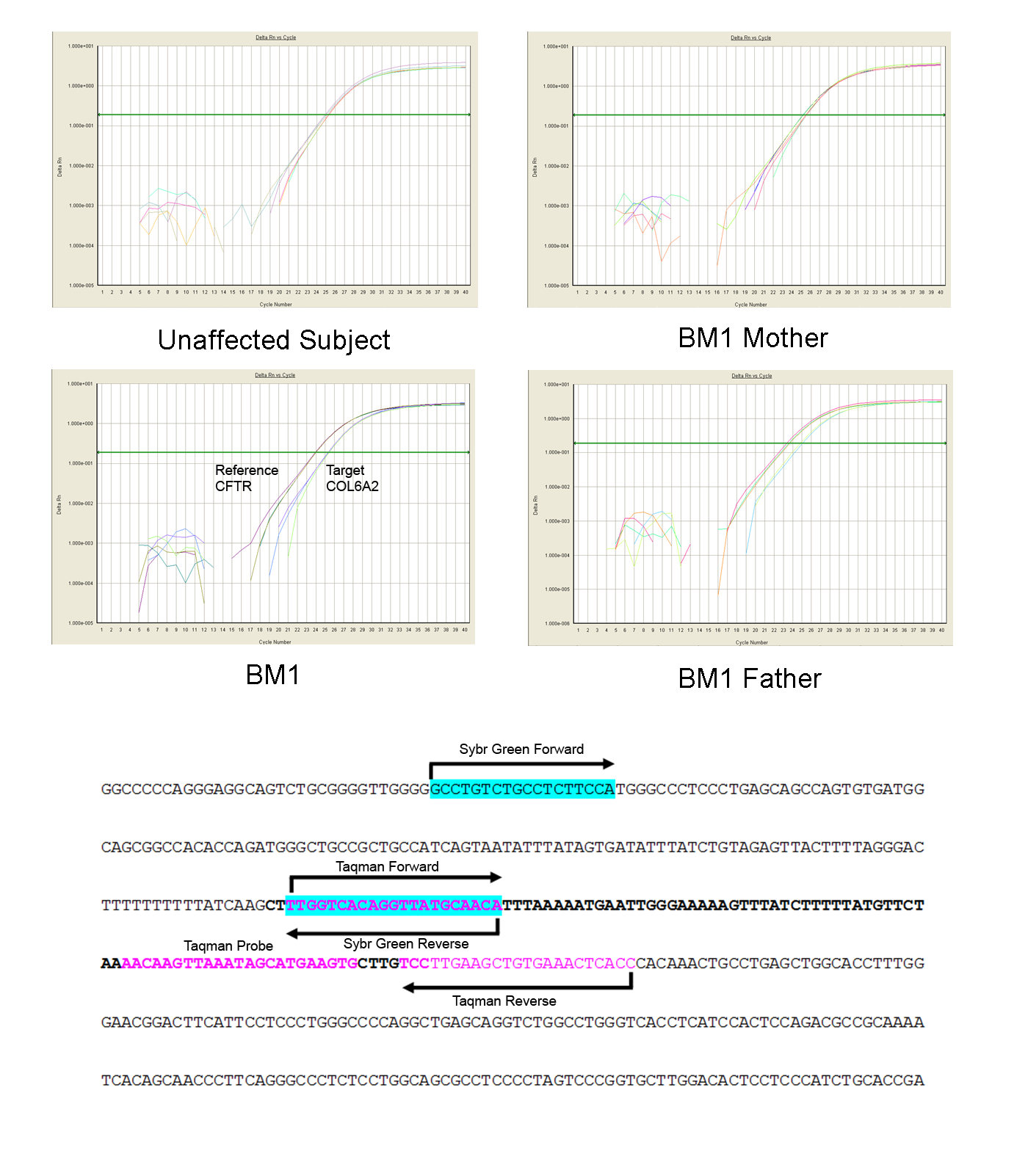

Supplement: Additional file 3 — Real Time PCR experiments confirming COL6A2 intron 1A deletion in BM Patient 1 and in the father. In the upper panel, the results obtained with an intron 1A specific TaqMan assay are shown. Red plots correspond to utilized reference gene (CFTR exon 15) whereas green plots refer to target sequence within intron 1A. The Ct (threshold cycle) values of the target sequence are in line with the reference in control sample (unaffected subject) and in proband's mother, whereas the target Ct values are higher than reference in BM Patient 1 and in the father, attesting the deletion (2-ΔΔCT values were 0.47 and 0.53 in BM Patient 1 and in the father respectively, whereas the 2-ΔΔCT value was 0.97 in the proband's mother). In the lower panel the position of the primers utilized in the SYBR green assay (blu) and of primers and probe utilized in the TaqMan assay (pink), are shown in respect to the deleted region (in bold). [file 1471-2350-11-44-S3.PNG]
